# Declining trends in early warning indicators for HIV drug resistance in Cameroon from 2008–2010: lessons and challenges for low-resource settings

**DOI:** 10.1186/1471-2458-13-308

**Published:** 2013-04-08

**Authors:** Joseph Fokam, Serge C Billong, Anne C ZK Bissek, Etienne Kembou, Pascal Milenge, Ibile Abessouguie, Armand S Nkwescheu, Zephirin Tsomo, Avelin F Aghokeng, Grace D Ngute, Peter M Ndumbe, Vittorio Colizzi, Jean BN Elat

**Affiliations:** 1 Chantal BIYA International Reference Centre (CIRCB) for research on HIV/AIDS prevention and management, Yaounde, Cameroon; 2 Faculty of Medicine and Biomedical Sciences (FMBS) of the University of Yaounde 1, Yaounde, Cameroon; 3 Central Technical Group (CTG), National AIDS Control Committee (NACC), Yaounde, Cameroon; 4 Department of Disease Control, Ministry of Public Health, Yaounde, Cameroon; 5 World Health Organisation (WHO) Afro, National Office, Yaounde, Cameroon; 6 Division of Operational Health Research (DROS), Ministry of Public Health, Yaounde, Cameroon; 7 Approved Treatment Centre, Yaoundé Central Hospital, Yaounde, Cameroon; 8 Virology Laboratory, Centre de Recherche en Maladies Emergentes et Ré-émergentes (CREMER)/IMPM/IRD, Yaounde, Cameroon; 9 Approved Treatment Centre (ATC), Yaounde General Hospital, Yaounde, Cameroon; 10 Faculty of Health Sciences (FHS), University of Buea, Buea, Cameroon; 11 UNESCO Biotechnology Chair, Department of Biological Sciences, University of Rome “Tor Vergata”, Rome, Italy

**Keywords:** Early warning indicator, HIV drug resistance, Surveillance and prevention, Cameroon

## Abstract

**Background:**

Rapid scale-up of antiretroviral therapy (ART) and limited access to genotyping assays in low-resource settings (LRS) are inevitably accompanied by an increasing risk of HIV drug resistance (HIVDR). The current study aims to evaluate early warning indicators (EWI) as an efficient strategy to limit the development and spread of preventable HIVDR in these settings, in order to sustain the performance of national antiretroviral therapy (ART) rollout programmes.

**Methods:**

Surveys were conducted in 2008, 2009 and 2010 within 10 Cameroonian ART clinics, based on five HIVDR EWIs: (1) Good prescribing practices; (2) Patient lost to follow-up; (3) Patient retention on first line ART; (4) On-time drug pick-up; (5) Continuous drug supply. Analysis was performed as per the World Health Organisation (WHO) protocol.

**Results:**

An overall decreasing performance of the national ART programme was observed from 2008 to 2010: EWI_1_ (100% to 70%); EWI_2_ (40% to 20%); EWI_3_ (70% to 0%); EWI_4_ (0% throughout); EWI_5_ (90% to 40%). Thus, prescribing practices (EWI_1_) were in conformity with national guidelines, while patient adherence (EWI_2_, EWI_3_, and EWI_4_) and drug supply (EWI_5_) were lower overtime; with a heavy workload (median ratio ≈1/64 staff/patients) and community disengagement observed all over the study sites.

**Conclusions:**

In order to limit risks of HIVDR emergence in poor settings like Cameroon, continuous drug supply, community empowerment to support adherence, and probably a reduction in workload by task shifting, are the potential urgent measures to be undertaken. Such evidence-based interventions, rapidly generated and less costly, would be relevant in limiting the spread of preventable HIVDR and in sustaining the performance of ART programmes in LRS.

## Background

Low- and middle-income countries had just over 8 million people receiving highly active antiretroviral therapy (HAART) by end of 2011, representing 54% [50–60%] coverage of eligible patients based on World Health Organisation (WHO) guidelines (CD4≤350 cells/μl) [1]. As compared to 2010 (<6 million) and 2003 (400,000), this coverage has been greatly favored by the rapid scale-up of antiretroviral therapy (ART), with sub-Saharan Africa being the main beneficiary (6.2 million people were receiving antiretroviral therapy in 2011, up from just 100,000 in 2003) [1,2]. In Cameroon, these efforts led to reductions in AIDS-associated morbidity and mortality, and a relative decrease in HIV prevalence (from 5.5% in 2004 to 4.3% in 2011), with close to 44.5% eligible patients on ART [3,4]. Furthermore, the number of ART clinics and of treated patients has increased overtime: 5 clinics for 116 (1.3%) eligible patients in 2002, to 145 clinics for 89,455 (36%) eligible patients in 2010 [4-6]. Since scale-up of ART is known to be associated with a high risk of HIV drug resistance (HIVDR), strategies to combat HIVDR are of public health priority in Cameroon [6-8]. Most importantly, with limited access to reference laboratory equipment (CD4 count, HIV viral load, HIVDR testing) and the use of drugs with low genetic barrier for resistance, HIVDR population-based surveillance and prevention are recommended to ensure long term efficacy of treatment guidelines [8-11]. As response to this crucial need, a national HIVDR working group (HIVDRWG) was created based on the WHO global HIVDR prevention and assessment strategy which includes the laboratory-based surveys of transmitted and acquired HIVDR, and monitoring of HIVDR early warning indicators (EWIs); with the guidelines being recently revised and updated in 2012 [12-17]. Surveys in Namibia, Malawi, South-Africa, and other African settings [18-24]; in Central American and Caribbean countries, as well as in Asia and Oceania (Papua New Guinea) [23-31], also supported the use of EWIs to evaluate the risk of ART failure and HIVDR emergence. Such evaluations use existing clinic- and pharmacy-based data on ART prescribing at the ART clinic [12,15,16]. Six strongly recommended and two optional EWIs are proposed by the WHO, among which ≥4 feasible EWIs should be chosen for an effective ART programme evaluation [15]. In this prospect, we aimed to evaluate the levels and trends of five WHO-strongly recommended EWIs, in order to identify potential strengths and weaknesses (i.e. gaps in service delivery that might inform policy changes to improve performance) of the national ART program and to target appropriate interventions that can optimize care and potentially minimize the risk of emerging resistant patterns that could compromise the commonly used antiretrovirals in the country. Of note, results presented in this study are based on WHO HIV drug resistance early warning indicator guidance [15], and not upon the updated 2012 guidance as presented in the latest WHO HIV drug resistance early warning indicator meeting report [16].

## Methods

### Study design and population

Using a retrospective review of clinical data from ART sites in Cameroon, five early warning indicators were assessed in 2008, 2009 and 2010 within 10 Cameroonian ART clinics. These ART clinics were chosen based on criteria among which: the number of years of experience on ART management (≥3 years), the frequency of new enrollments on ART per quarter (i.e. ≥30 newly enrolled patients on ART every 3 months), the geographical locations of the ART clinic over the national territory, and finally on convenience amongst sites meeting these criteria. At the beginning of the study in 2008, 133 ART clinics {24 approved treatment centers [ATCs] and 109 HIV management units: [HMUs]} existed nationwide, among which 89 were already operational since 2005. Thus, based on the above mentioned eligibility criteria (≥3 years experience), our study coverage represented a proportion of ≈11% (10/89) of the entire national ART clinics.

The five EWIs were selected and defined according to the WHO criteria [12,15]:

– EWI_1_: “*Good ARV prescribing practices*” (Percentage of patients initiated on an appropriate first line ARV drug regimen). Numerator: Number of individuals initiating ART at the site who are prescribed a standard or otherwise appropriate first-line regimen during the selected time period. These are first line regimens recommended by the country national guidelines, and exclude dual- or mono-therapy. Denominator: Number of individuals starting ART during the selected time period. The acceptable target performance: 100%.

– EWI_2_: “*Patient lost to follow*-*up*” (Percentage of patient lost to follow-up after 12 months of enrolment to ART). Numerator: Number of individuals initiating ART in a selected time period that were not seen at the clinic or pharmacy ≥90 days after the date of their last missed appointment or drug pick-up that occurred within their first 12-months of ART, and who are not known to have transferred out or died. Denominator: Number of individuals starting ART during a selected time period. The acceptable target performance ≤20%.

– EWI_3_: “*Patient retention on appropriate first line ART*” (Percentage of patient retention on appropriate first line ART after 12 month of treatment). Numerator: Number of individuals initiating first-line ART during a selected period of time who are, 12 months from ART start, still on first-line ART (this includes substitutions of one appropriate first-line regimen for another, but not substitutions of dual- or mono-therapy or an inappropriate three-drug regimen). Denominator: Number of individuals starting ART during a selected time period or, in sites where data are available, that number minus the number of individuals starting ART in that time period who transferred out during the 12 months after starting ART. Individuals who died, stopped ART, switched to second-line ART, or were lost to follow-up must be included in the denominator. The acceptable target performance ≥70%.

– EWI_4_: “*On*-*time ARV drug pick*-*up*” (Percentage of on-time ARV drug pick-up by the patient). Numerator: number of individuals who have picked up all their prescribed ARV drugs on time during the selected time period. This EWI uses a sample of all patients on ART, and follows them for two drug pick-ups after baseline pick-up. Denominator: number of individuals classified as "on ARV drugs" during the selected time period. The acceptable target performance ≥90%.

– EWI_5_: “*Drug supply continuity*” (Percentage of ARV drug supply continuity at the site pharmacy). Numerator: Number of months or quarters in the year in which there were no ARV drug stock outages for any ARVs in any of the standard ART regimens supplied by the site or the pharmacy at which the site's patients pick up ARV drugs. Denominator: 12 months. The acceptable target performance: 100%.

Sample size for each ART site was calculated based on the WHO guidance. In detail, for the first year (2008), considered as the ever first or pilot phase for such study in the country, a sample size of thirty (30) patients per site was required. Patient sampling for the two-other yearly evaluations (2009–2010) was then based on the total number of new enrollments at ART during the study year, with respect to the following WHO-requirements shown in Table 1. This sampling method derived from the following formula: N = (Z^2^ x P x Q)/d^2^; with N being the minimum sample size, Z the confidence interval at 95% (Z=1.96), P the expected percentage of retention on ART, Q as 1- P, and D the precision at a degree of accuracy (d=7%) [12,15].

**Table 1:** **WHO**-**sampling method**[12,15]

**Number of newly enrolled patients on ART per year**	**Required sample size**
1-75	Recruit all
76-110	75
111-199	100
200-250	110
251-299	120
300-350	130
351-400	135
401-450	140
451-550	145
551-700	155
701-850	160
851-1600	175
1601-2150	175
2151-4340	200
4341-5670	210
5671-10,000	215
>10,000	WHO advice required

*WHO*: World Health Organization.

### Data collection, validation and analysis

Description of each ART site was carried out as per WHO suggested “site profile”, which includes the category of the site (ATC or HMU), the geographical localization, years of experience in ART management, the number of patients on ART, number and category of trained staff, availability of treatment guidelines, and availability of first/second line ARV drugs.

Data were collected using WHO data abstraction tools (standardized sheets) among patients newly enrolled on ART and followed-up ≥one year. Briefly, the first three EWIs were obtained by abstraction of cumulative cohort data from ART registers, while the two other EWIs were abstracted from the pharmacy register. In order to ensure reliability in the quality of data, data verification/validation was performed using 20% of data from the medical records. Validation was a two levels process: firstly on-site (by source verification in ART and pharmacy registers, against the WHO standardized data collection sheets), and then secondly at the central level (by verification between the standardized collection sheet against the WHO-electronic routine data quality assessment tool: “*RDQA*”). The levels of EWIs were obtained each year, and the trends were evaluated through, a chronological analysis, using these 10 ART clinics enrolled continuously throughout the three years (2008, 2009 and 2010), and statistically significance overtime was analyzed for the performance of each EWI using the Fisher exact test, with a p-value <0.05 considered as a statistically significant difference. EWI attaining the acceptable target was classified as “*Good Performance*”, while EWI unable to reach the target was classified as “*Poor Performance*”.

### Ethical considerations

With respect to the Helsinki Declaration for research carried out on humans (including human material or human data), ethical approval was obtained from the National Ethics Committee (Authorization N°034/NEC/SE), Yaounde, Cameroon. Confidentiality was ensured during data abstraction by the use of identification codes. Since the study focused on ART site performance rather than individual patient, informed consent from patients was not required. For on-site knowledge transfer and owning of EWI activities to staff, heads of the ART clinics and their respective data managers were preliminarily trained on HIVDR EWIs and on the methodology for data collection, analysis and interpretation.

## Results

### Description of the ART clinics

Data validation was successful for all the 10 ART clinics throughout the three years (2008, 2009 and 2010). These ART clinics were geographically distributed in four different regions of the country, among which four sites from the Centre region (Yaounde General Hospital, National Social Welfare Hospital, Yaounde Central Hospital, and Yaounde Jamot Hospital); three sites from the Littoral region (Douala General Hospital, Laquintinie Hospital Douala, and Nylon District Hospital); two sites from the North-west region (Bamenda Regional Hospital, and Polyclinic Mezam Bamenda); one site from the South-west region (Limbe Regional Hospital).

These ART clinics had a median working experience in the HIV/AIDS therapeutic management of 6.5 years [Interquartile range, IQR: 5–7]. The national ART guidelines were available in all the sites, as well as treatment protocols for first line ARV drugs; second line drug regimens were available solely in ATCs. ARV drugs were provided at a pharmacy within the clinic; and drug stocks were run using designed sheets.

Throughout the study, each of the clinics had more than 30 patients newly enrolled on ART quarterly (i.e. in every three months period). By the end point (i.e. in 2010), a total 27,826 patients were on ART in the entire study clinics, and staffs (physicians, nurses, pharmacists/clerks, biologists/laboratory technicians, counselors, data managers, community relay agents) were all trained on the routine management of people living with HIV. The staff-patient distribution per site was as follows: Yaounde General Hospital (1,542 patients for 24 staffs; ≈64/1 ratio), National Social Welfare Hospital (1,247 patients for 178 staffs; ≈6/1 ratio), Yaounde Central Hospital (6,031 patients for 43 staffs; ≈140/1 ratio), and Yaounde Jamot Hospital (1,610 patients for 33 staffs; ≈49/1 ratio), Douala General Hospital (2,049 patients for 34 staffs; ≈61/1 ratio), Laquintinie Hospital Douala (3,831 patients, staffs number not provided), Nylon District Hospital (2,800 for 34 staffs; ≈83/1 ratio), Bamenda Regional Hospital (4,032 patients for 31 staffs; ≈130/1 ratio), Polyclinic Mezam Bamenda (2,100 patients for 18 staffs; ≈117/1 ratio), and Limbe Regional Hospital (2584 patients for 191 staffs; ≈14/1 ratio). Thus, this resulted to an overall median staff-patient ratio of ≈1/64 [Interquartile range (IQR): 1/49 – 1/117], indicative of a heavy workload; interestingly, the only ART site with ≥50% good performance over the three years survey (H. CNPS: 60% good performance i.e. 9/15, as shown in Table 1) had the lowest workload (1/6 staff/patient ratio). An overview distribution of patients per staff category was as follows: medical doctor (1:267 patients); nurse (1:74 patients); biologists/laboratory technicians (1:434 patients); pharmacist/pharmacy clerk (1:694 patients); counselor (1:434 patients); community relay agent (1:323 patients); and data manager (1:1387 patients).

### Performances of EWIs in 2008, 2009, and 2010

For EWI_1_, the overall yearly performance moved from 100% (10/10) in 2008, 80% (8/10) in 2009, to 70% (7/10) in 2010; without any statistically difference overtime (p= 0.47). Of note, the few sites with suboptimal performances had each a target of 98-99%, which is much closed to the required performance of 100%. No dual- or mono-therapy was reported in any of the studied clinics.

For EWI_2_, only 40% (4/10) reached the required target (≤20%) in 2008 against 20% (2/10) in 2009 and 20% (2/10) in 2010; revealing an increasing rate of patients lost to follow-up between the first two years, followed by stable rate; still without any statistically significant difference over time (p=0.82).

For EWI_3_, 70% (7/10) of sites reached the required performance target (≥70%) in 2008, against 10% (1/10) and 0% (0/10) in 2009 and 2010, respectively; describing an overall decreasing performance in terms of patient retention on first line ART, with a statistically significant difference overtime (p= 0.0049).

For EWI_4_, the rates of patients with on-time drug pick-up were extremely below the required target (≥90%) during each of the three years. In details, the median performances varied from 36.5% [IQR: 28-47%] in 2008, to 15% [IQR: 10-21%] in 2009, and to 33% [IQR: 21-37%] in 2010; with a statistically significant difference overtime (p= 0.0132).

For EWI_5_, drug availability, also known as continuity in drug supply, decreased significantly (p= 0.006) from 90% (9/10) in 2008 to 20% (2/10) in 2009, then later reported a non-significant increment (p=0.625) to 40% (4/10) in 2010. Interestingly, throughout the three years, the overall performance showed a significant drop down in drug supply overtime (p=0.023).

**Figure 1:**
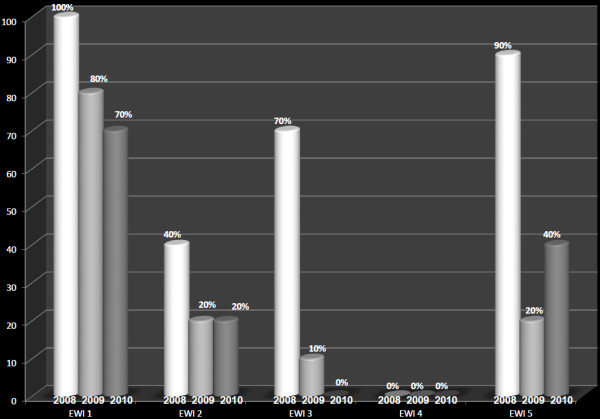
**Trends of EWIs from 2008**–**2010. **EWI_1_: Early Warning Indicator 1, EWI_2_: Early Warning Indicator 2, EWI_3_: Early Warning Indicator 3, EWI_4_: Early Warning Indicator 4, EWI_5_: Early Warning Indicator 5. For each EWI, the graphs represent the national performance in 2008, 2009, and 2010, respectively.

The overall trends (from 2008–2010) of the five HIVDR EWIs under study are reported in Figure 1, showing the majority of clinics having optimal performances on prescribing practices (EWI_1_) without any statistically significance, while the other four EWIs revealed suboptimal and decreasing performances overtime, most often (3/4) with significant differences overtime. The detail performance of EWIs, during each of the three years, and in each of the 10 surveyed ART clinics, is reported in Table 2.

**Table 2:** **Site performances of EWI from 2008**-**2010**

**EWI per year ART sites**	**EWI 1**: **Target**=**100% (n**/**total)**			**EWI 2**: **Target**≤**20% (n/total)**			**EWI 3**: **Target**≥**70% (n/total)**			**EWI 4**: **Target**≥**90% (n/total)**			**EWI 5**: **Target=100% (n**/**total)**			**Overall performance per site % (n/total)**
	**2008**	**2009**	**2010**	**2008**	**2009**	**2010**	**2008**	**2009**	**2010**	**2008**	**2009**	**2010**	**2008**	**2009**	**2010**	
Y.G.H	**100**%	**100**%	**100**%	37%	39%	35%	63%	47%	56%	47%	19%	29%	92%	33%	58%	20% (3/15)
	(**30**/**30**)	(**200**/**200**)	(**135**/**135**)	(11/30)	(77/198)	(47/135)	(19/30)	(94/200)	(76/135)	(14/30)	(37/192)	(39/135)	(11/12)	(4/12)	(7/12)	
H. CNPS	**100**%	**100**%	99%	**10**%	**20**%	**20**%	**90**%	**70**%	56%	30%	39%	37%	**100**%	50%	**100**%	60% (9/15)
	(**30**/**30**)	(**200**/**200**)	(158/160)	(**3**/**30**)	(**39**/**194**)	(**20**/**132**)	(**27**/**30**)	(**133**/**193**)	(90/160)	(9/30)	(78/200)	(58/157)	(**12**/**12**)	(6/12)	(**12**/**12**)	
Y.C.H	**100**%	**100**%	**100**%	27%	31%	39%	**77**%	38%	55%	28%	12%	32%	**100**%	92%	67%	33% (5/15)
	(**30**/**30**)	(**200**/**200**)	(**180**/**180**)	(8/30)	(62/200)	(64/165)	(**23**/**30**)	(76/200)	(94/170)	(8/30)	(24/200)	(58/180)	(**12**/**12**)	(11/12)	(8/12)	
Y.J.H	**100**%	**100**%	**100**%	**10**%	46%	43%	**77**%	27%	50%	3%	15%	56%	**100**%	75%	75%	40% (6/15)
	(**30**/**30**)	(**200**/**200**)	(**155**/**155**)	(**3**/**30**)	(86/186)	(60/139)	(**23**/**30**)	(51/186)	(73/145)	(1/30)	(29/196)	(86/153)	(**12**/**12**)	(9/12)	(9/12)	
D.G.H	**100**%	**100**%	99%	40%	41%	38%	60%	47%	36%	50%	57%	50%	**100**%	25%	**100**%	27% (4/15)
	(**30**/**30**)	(**188**/**188**)	(158/160)	(12/30)	(76/185)	(60/160)	(18/30)	(89/188)	(58/159)	(15/30)	(113/199)	(74/147)	(**12**/**12**)	(3/12)	(**12**/**12**)	
L.H.D	**100**%	**100**%	99%	37%	29%	33%	63%	66%	60%	43%	21%	34%	**100**%	**100**%	**100**%	33% (5/15)
	(**30**/**30**)	(**200**/**200**)	(158/160)	(11/30)	(58/200)	(53/160)	(19/30)	(132/200)	(93/156)	(13/30)	(41/194)	(54/160)	(**12**/**12**)	(**12**/**12**)	(**12**/**12**)	
N.D.H	**100**%	**100**%	**100**%	33%	**15**%	58%	**70**%	35%	44%	30%	10%	12%	**100**%	50%	**100**%	47% (7/15)
	(**30**/**30**)	(**200**/**200**)	(**175**/**175**)	(10/30)	(**25**/**162**)	(101/175)	(**21**/**30**)	(127/194)	(70/159)	(9/30)	(19/200)	(20/171)	(**12**/**12**)	(6/12)	(**12**/**12**)	
L.R.H	**100**%	99%	**100**%	27%	40%	**19**%	**73**%	57%	44%	13%	2%	0%	**100**%	**100**%	42%	40% (6/15)
	(**30**/**30**)	(196/198)	(**160**/**160**)	(8/30)	(80/200)	(**30**/**160**)	(**22**/**30**)	(113/199)	(68/153)	(4/30)	(3/199)	(0/160)	(**12**/**12**)	(**12**/**12**)	(5/12)	
B.R.H	**100**%	**100**%	**100**%	**20**%	97%	41%	**70**%	1%	49%	43%	15%	34%	**100**%	50%	17%	40% (6/15)
	(**30**/**30**)	(**200**/**200**)	(**172**/**172**)	(**6**/**30**)	(193/198)	(68/167)	(**21**/**30**)	(2/200)	(82/167)	(13/30)	(29/197)	(50/146)	(**12**/**12**)	(6/12)	(2/12)	
P.M.B	**100**%	98%	**100**%	**10**%	32%	25%	**87**%	34%	53%	77%	9%	21%	**100**%	42%	42%	33% (5/15)
	(**30**/**30**)	(**176**/**180**)	(**114**/**114**)	(**3**/**30**)	(54/170)	(28/114)	(**26**/**30**)	(60/180)	(62/117)	(23/30)	(19/211)	(26/125)	(**12**/**12**)	(5/12)	(5/12)	

Legend Table 1: in “**Bold**” are performances that meet the required target.

**Y**.**G**.**H**: Yaounde General Hospital; **H**. **CNPS**: National Social Welfare Hospital; **Y**.**C**.**H**: Yaounde Central Hospital; **Y**.**J**.**H**: Yaounde Jamot Hospital; **D**.**G**.**H**: Douala General Hospital; **L**.**H**.**D**: Laquintinie Hospital Douala; **N**.**D**.**H**: Nylon District Hospital; **L**.**R**.**H**: Limbe Regional Hospital; **B**.**R**.**H**: Bamenda Regional Hospital; **P**.**M**.**B**: Polyclinic Mezam Bamenda.

## Discussions

The increasing need to control HIVDR in low-resource settings (LRS) requires the implementation of efficient and rapid approaches to limit the emergence of preventable HIVDR, among which a population-based survey, such as EWI evaluations, could yield relevant evidences based-recommendations for best clinical practices. Overall, this is a paper on a topic of high importance in HIV medicine, transmitted drug resistance, as well as the methods for detecting it in a LRS where genotyping is not available, and remains challenging and clinically important issues to all the stakeholders who are involved in the global scale up of ART, particularly in Africa.

Physicians’ prescribing practices (EWI_1_) remained nationwide in conformity with the national guidelines. Interestingly, even sites with the 98-99% performances for EWI_1_ had no cases of dual- or mono-ARV prescriptions, which are more likely to select for drug resistant HIV rather than an inappropriate prescribing of drugs such as boosted protease inhibitors, abacavir, or tenofovir (which may not be appearing on several guidelines) which likely have less significance related to HIVDR. This good practice was mainly due to the use of standardized ART regimens and the institution of a consortium agreement (therapeutic committee) for every treatment initiation. This is a weekly consortium body made up of health staff all trained in HIV/AIDS management (medical doctors, nurses, medical biologists/laboratory technicians, medical counselors, pharmacists/pharmacy clerks, data manager and/or community relay agents; a practice which merits to be encouraged. Globally, 75% of clinics monitored worldwide met the WHO-recommended target on prescribing practices [16]. Specifically, other African countries (Malawi, Namibia, and others) also reported good practices [18-24], while poor performances were observed out of Africa (Central American, Caribbean, Asian, Oceanic and Western Pacific: from 38 to 75%) [23-31]. Lack of evaluation overtime in the above mentioned studies could not enable in-depth comparison to ours. Still, the use of a therapeutic committee as well as continuous training may be vital practices to maintain and/or improve good ARV prescriptions.

The high and increasing rates of lost-to-follow-up are indicative of a growing rate of patients harboring potential drug resistant viruses within the national context, which in turns supports a growing risk of HIVDR emergence in Cameroon. These would have been favored by the observed heavy workload, and also probably due to termination of working contracts for community relay agents (CRA). Thus, task shifting (from medical doctors to nurses), and probably community empowerment (by allocating resources to CRAs), as well as free consultations and reduced laboratory costs in such poor settings, may help in reducing the rate of missing patients. Indeed, the numbers of required health care workers to provide ART in LRS (1–2 physicians/1000 patients, 2–7 nurses/1000 patients, <1-3 pharmacy staff/1000 patients, and wider ranges for other health-allied staffs) estimated by Hirschhorn *et al*. [32], was based on the WHO-3by5 target (i.e. treatment of 5 million patients by 2005), and currently need to be revised in order to match the growing ART coverage (presently >8 millions) [1]. With restriction to the WHO-protocol, a direct evidence-based evaluation has not yet been conducted to investigate on the real impact of CRA’s disengagement on lost to follow-up. Globally, 69% of clinics monitored worldwide met the WHO-recommended target [16]. Particularly, in other African countries, lost-to-follow-up is also challenging (40 to 75%) [18-24], whereas out of Africa higher performances were recorded (54 to 100%) [23-31]. Furthermore, the impact of distance to the clinic, waiting time prior to medical consultation, stigma and patient educational level, are factors that may help in reducing lost patients [33]. Indeed, ART uptake has been negatively associated with distance from the nearest primary healthcare [34,35], thus indicating a possible need for creating new ART clinics to foster adherence.

The gradual poor retention of patients on first line ART after 12 months ART may be indicating a rapid switch to second line regimens that could be explained by numerous other factors (wider availability of HIV Viral Load testing, greater clinician experience/awareness on identifying treatment failure, a preexisting drug resistant mutation, etc.). Further studies are therefore needed to determine the time-to-treatment, to implement measures toward long term efficacy of first line ART and to limit events of inappropriate switch to second line ART in these settings. Furthermore, failure to be on first line therapy one year after initiation might in fact not necessarily be a negative indicator, since it might indicate a correct and necessary treatment switch to second line. This observation makes questionable the overall utility of EWI_3_, which would likely need to be revised for better utility in future. Globally, 67% of clinics monitored worldwide met the WHO-recommended target [16]. More interestingly, our performances in 2008 (70%) were similar to those found in other African settings (among which Malawi: 53% and Namibia: 67%) [18-24], while higher performances (62-90%) were recorded out of Africa [23-31]. Since first line ARV drugs (≈$100/patient/year) are about 4 times less costly than second line, an effective retention on first line is economical [36] and may regulate the current fast switch to second line ARV (2% of total patients on ART in 2006, to 13% of total patients on ART in 2009) in Cameroon [4]. These analyses are consistent with the different HIVDR rates to NRTI and NNRTI in 2003 (before ART scale-up: 5.6%M184V and 6.1%Y181C) and thereafter (following ART scale-up: 16.3%M184V and 63.7%Y181C) [37]. Despite the higher rate of transmitted HIVDR in Latin America (12-20%), sub-Saharan Africa, though with only 4-9% of transmitted HIVDR, is experiencing a rapid scale-up of ART associated with an increasing rate of transmitted HIVDR (38% increasing risk of HIVDR per year): this important fact urgently needs a regular HIVDR surveillance system [14,16,38].

The overall delay in drug pick-up strongly supports a national revision of the community engagement strategy to support patient adherence to ART programmes. This poor/decreasing performance in drug pick-up (EWI_4_), together with EWI_2 and 3,_ clearly placed patient non-adherence as the main factor with high risks of HIVDR development and spread within the community. However, it should be noted that EWI_4_ itself seems problematic and may not be entirely practical to address adherence issues. Indeed, in our setting, clients may present one-to-two day(s) after their required drug pick-up appointment date and still maintain 100% ART adherent, due to availability of remaining pills from the previous appointment. Thus, as for EWI_3_, the overall utility of EWI_4_ may also be brought into question, and would likely require expert review or reconsideration. Thus, the low scores found in our study are partly explained by the rigidity of this indicator, which has further being recently modified in the 2012 HIVDR report [16]. Globally, 70% of clinics monitored worldwide met the WHO-recommended target for timely drug keep-up [16]. Detail analysis also reports lower timely drug pick-ups in other African settings (between 17% and 41%) [18-24], against settings out of Africa (73-100%) [23-31]. Thus, in African AIDS programmes, issues such as “time spent by patient for pharmacy service”, “service quality rendered by pharmacy staff”, “pharmacy localization”, may be partly explained by delays in drug pick-up. As suggested by El-Khatib et al. in 2011, adherence to drug-refill (pill count) may also be a useful EWI of virologic and immunologic failure on first-line ART in African settings [23].

The gradual poor performances in drug supply present EWI_5_ as the main programmatic setback in the national ART performance. Despite the provision of alternative ART (by replacing shortage of efavirenz with nevirapine or lopinavir/ritonavir) in case of drug discontinuity, stock outs still negatively impact patient adherence with non-negligible risks of HIVDR due to potential suboptimal drug levels. Drug supply machinery should be urgently revised. Our findings could be strengthened by conducting further investigations to as to why these stock outs are occurring; targeting specifically the supply lines, trade agreements specific to Cameroon, land versus air versus sea route issues, or in-country distribution network problems. Globally, 65% of clinics monitored worldwide met the WHO-recommended target for drug supply. Geographically, the procurement systems were successful (100%) in Malawi (cross-sectional study) [18], and with an increasing performance in the Caribbean (31.3%-94%, from 2007–2009) [23,24]. Aggregated data showed poorer performance in sub-Saharan Africa (42%-47%), as compared to non-African countries (32-90%) [20-31]. Thus, African settings are more in need of further investigations to improve their drug supply system.

### Lessons learnt from the three year survey

This study has effectively identified some strengths and weakness of the national ART programme, amongst which the encouraging prescribing practices, and corrective measures been addressed for other EWIs. Indeed, the performance of these later EWIs has decreased over time rather than staying steady or improving (as would have been hoped). Thus, this result is also very interesting, and if taken at face value, is a serious indictment of the overall implementation strategies underway in Cameroon, and a crucial cause for concern for generation of drug resistance. Therefore, tasks shifting/decentralization (to alleviate the heavy workload) and community re-empowerment are underway [4,39]. A specific algorithm (in form of posters and hand-outs) presenting overall performances and addressing adapted corrective actions, has been provided to guide healthcare providers working at the ART clinic (see Additional file 1). Also, advocacy, addressed to health authorities (ministry and heads of health facilities), boosted the implementation of the above corrective measures. Additionally, a bottom-to-top approach, through consideration of associations of people living with HIV, would allow patients to participate as key players in the success of the national ART performance [33,35,36,39]. Site supervision is also essential for an effective integration of HIVDR activities in the routine clinical practices, to make EWI an instrumental in prioritizing measures and allocating resources for clinics. Our analyses suggest that ART programmes in other LRS may be experiencing similar declining performances, and thus need relevant measures.

### Challenges resulting from our findings

A successful and sustainable ART programme performance should be accompanied by scalable EWI survey in the country; the major setback relies on the regular availability of the required resources [12]. Secondly, despite the availability of external support, additional fund raising is still needed to optimize patient healthcare. Continuous staff training is essential to sustain good practices; brain drain makes the heavy workload persistent, thereby calling for policies to train and retain qualified personnel, especially with the need/creation of new ART clinics in the country to ensure scaling-up of the treatment programmes [36]. More importantly, recent studies in Cameroon showed low to moderate levels of transmitted HIVDR [40], and increasing levels of acquired HIVDR after 12 and 24 months [41], thus predicting growing risks of treatment failure and HIVDR to the commonly used drugs, due to a broad range of factors [35,39]. Without attempting to create a direct temporal relationship, the increasing rate of transmitted and acquired HIVDR in Cameroon, alongside the increasing/widely availability of ARV treatment, supports also a growing need of affordable viral load and HIVDR testing, and a more regular surveillance of HIVDR in this country [8,38]. Pediatric HIVDR surveillance is of prime importance, and needs to be implemented [42]. Of note, as observed in other countries, *Fokam et al*. also found low (4.9%) and high (90%) rates of HIVDR among drug-naïve and first-line ART failing children in Cameroon, respectively [43]. Finally, the emerging HIV co-infections with tuberculosis, malaria, viral hepatitis, require setting-up an antimicrobial drug resistance strategic plan and working group to preserve active drugs for the next generation [44,45].

Despite restrictions to WHO-standards, our study limitations could not allow greater/meaningful statistical analysis, due to the limited number of sampled ART sites. Furthermore, difficulties in evaluating other factors that could potentially affect ART performances (disengagement of CRAs, task shifting, distance to clinic, stigma/discrimination, educational level, bottle nets in the drug supply system, etc.) also limited the strengths of our recommendations.

## Conclusions

The extended use of ART in developing countries warrants a thorough understanding of all factors contributing to the success of national ART programmes. In the Cameroonian setting, patient adherence, drug stock outs, a potential community disengagement and a probable heavy workload, appear as major factors favoring an increasing risk of HIVDR emergence.

Use of EWI is an effective approach to identify both patient and programmatic factors favoring the risks of HIVDR at the clinics and at the national levels. With its simplicity, low cost, and rapid evidence-based policy-making, such surveys would be useful for a successful scale-up of ART in LRS, which in turns contribute to the global HIVDR prevention and surveillance strategy.

## Competing interests

Authors declare that they have no financial, personal, or professional interests that could be construed to have influenced the paper.

## Authors’ contributions

Conceived and designed the study: GDN, SCB, JF, ACZKB, EK, PM, IA, ASN, AFA, PMN, VC, JBNE. Acquired the data: ZT, SCB, JF, EK, PM, IA, ASN, GDN. Analyzed and interpreted the data: JF, SCB, EK, PM, ZT, GDN, IA, JBNE. Drafted the manuscript: JF, SCB, EK, PM, GDN, JBNE. Revised the manuscript: ACZB, IA, ASN, ZT, AFA, PMN, VC. Approved the final version of the manuscript: SCB, JF, ACZB, EK, PM, IA, ASN, AFA, ZT, GDN, PMN, VC, JBNE. All authors read and approved the final manuscript.

## Pre-publication history

The pre-publication history for this paper can be accessed here:

http://www.biomedcentral.com/1471-2458/13/308/prepub

## Supplementary Material

Additional file 1 Recommendations to limit HIV Drug Resistance in Cameroon. Click here for file
