# Supplementary figures and images for: Declining trends in early warning indicators for HIV drug resistance in Cameroon from 2008–2010: lessons and challenges for low-resource settings
Source: BMC Public Health. 2013 Apr 8;13:308. doi: 10.1186/1471-2458-13-308 (PMC3627634; doi:10.1186/1471-2458-13-308)

## Slide 1
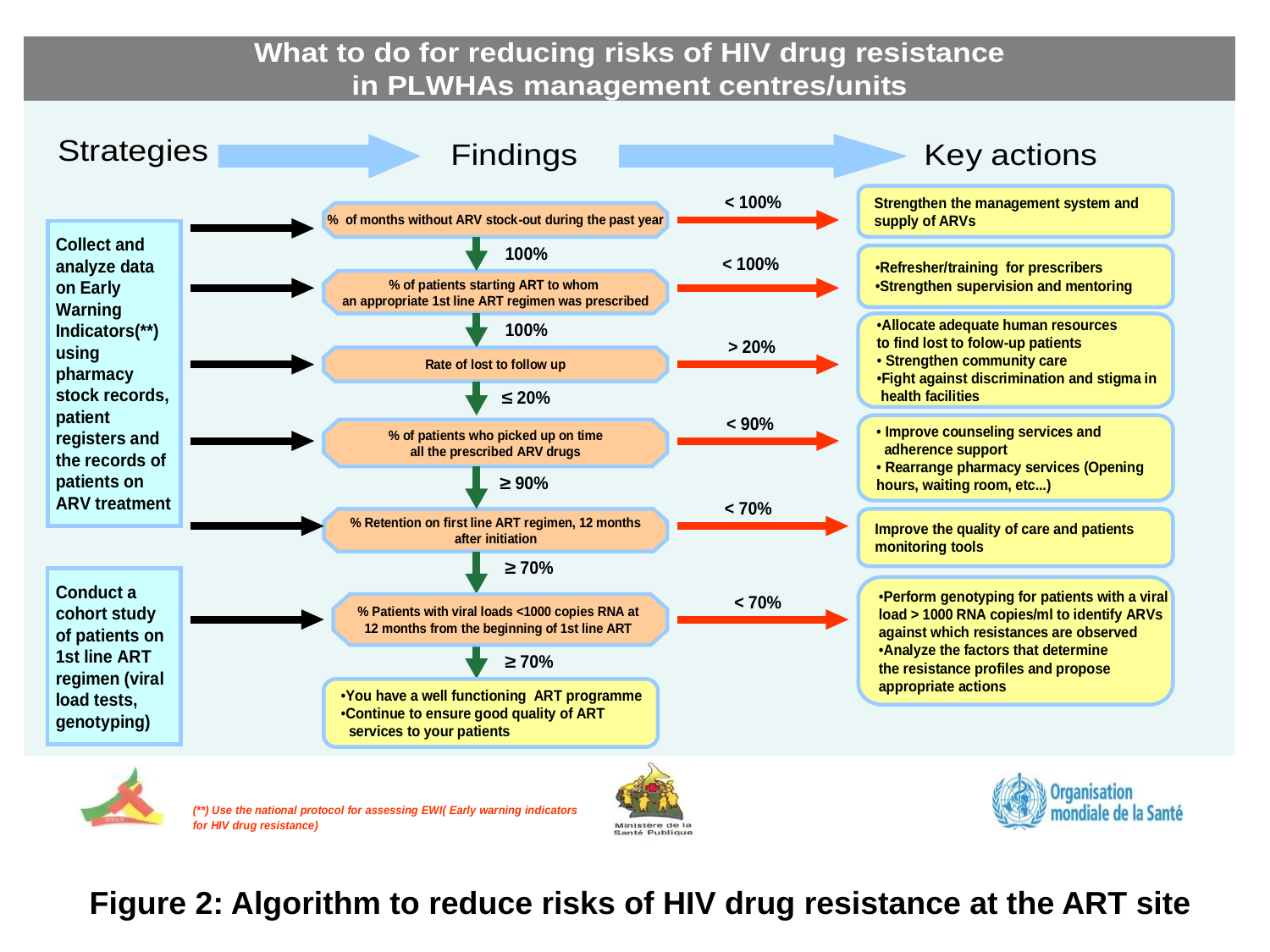

# Figure 2: Algorithm to reduce risks of HIV drug resistance at the ART site

Supplement: Additional file 1 — Recommendations to limit HIV Drug Resistance in Cameroon. [file 1471-2458-13-308-S1.ppt]
